# Intraarticular leukocyte-poor platelet-rich plasma injection is more effective than intraarticular hyaluronic acid injection in the treatment of knee osteoarthritis: a systematic review and meta-analysis of 12 randomized controlled trials

**DOI:** 10.1186/s43019-025-00266-5

**Published:** 2025-03-28

**Authors:** Yu-Ning Peng, Yu-Hsiang Peng, Jean-Lon Chen, Carl P. C. Chen

**Affiliations:** 1 Department of Physical Medicine and Rehabilitation, Chang Gung Memorial Hospital at Linkou, Chang Gung University, Guishan District, Taoyuan City, Taiwan; 2 Department of Physical Medicine and Rehabilitation, Chang Gung Memorial Hospital 5 at Taoyuan, Chang Gung University, Fu-Hsin St., Kwei-Shan, Guishan District, Taoyuan City, 333 Taiwan; 3 Department of Medicine, MacKay Medical College, Sanzhi District, New Taipei City, Taiwan

**Keywords:** Knee, Osteoarthritis, Leukocyte-poor platelet-rich plasma, Leukocyte-rich platelet-rich plasma, Hyaluronic acid

## Abstract

**Purpose:**

We aim to compare the clinical effects of intraarticular leukocyte-poor platelet-rich plasma (LP-PRP) injection with those of intraarticular hyaluronic acid (HA) injection in adult patients with knee osteoarthritis.

**Methods:**

Two authors independently reviewed databases, including PubMed, Web of Science, and the Cochrane Library. Only randomized controlled trials (RCTs) were included in our meta-analysis. Western Ontario and McMaster Universities Arthritis Index (WOMAC) scores (WOMAC total, pain, stiffness, and physical function scores), visual analog scale (VAS) scores, EQ-VAS scores, International Knee Documentation Committee (IKDC) scores, and adverse events were used as outcome measurements to evaluate the efficacy of LP-PRP and HA treatment.

**Results:**

After screening 377 potential articles, 12 RCTs were included in this systemic review and meta-analysis. The WOMAC total scores and WOMAC physical function scores of the LP-PRP group were better than those of the HA group at 6 and 12 months. VAS scores of the LP-PRP group were better than those of the HA group at 3, 6, and 12 months. The LP-PRP group showed a better outcome of IKDC scores than the HA group at 6 months. There was no significant difference in adverse events between the LP-PRP and HA groups.

**Conclusion:**

Intraarticular injections of LP-PRP showed better overall outcomes, such as WOMAC total scores, WOMAC physical function scores, VAS scores, and IKDC scores, compared with HA for adult patients with knee osteoarthritis at 6- and 12-month follow-up periods. Also, LP-PRP showed better pain relief compared with HA at 3-, 6-, and 12-month follow-up periods. Intraarticular LP-PRP improves pain relief and overall outcomes in patients with knee osteoarthritis.

## Introduction

Knee osteoarthritis (OA) stands as a prevalent chronic arthritic condition among the elderly population, characterized by the gradual degeneration of cartilage and subsequent joint space narrowing [1]. Conventional pharmacological interventions targeting symptomatic knee OA predominantly entail oral administration of nonsteroidal anti-inflammatory drugs (NSAIDs), acetaminophen, glucosamine, and chondroitin. Nevertheless, it is worth noting that the utilization of NSAIDs and analgesics is frequently associated with adverse effects. By contrast, as a minimally invasive therapy, it is concluded that intraarticular (IA) injections of autologous platelet-rich plasma (PRP) and hyaluronic acid (HA) serve as a more suitable and effective nonsurgical treatment of knee OA [2]. HA is a natural glycosaminoglycan generated by chondrocytes, synoviocytes, and fibroblasts. By providing the viscoelastic characteristics of the knee joint and increasing the lubrication of the articular surface, HA has been demonstrated to improve joint function and relieve pain in knee, hip, and ankle OA [3]. PRP is an autologous blood product of highly concentrated platelets containing growth factors that can modulate inflammation and improve angiogenesis in the treated area [4]. PRP modifies the interactions between different cell phenotypes. In addition, PRP is drawing interest in promoting myogenic differentiation without profibrotic factors such as TGF-β1 [5]. Moreover, the biological properties of PRP vary for each individual on the basis of internal and external factors such as age, immune status, metabolic diseases, and medications [6]. Before a PRP injection, it is important to discontinue NSAIDs, anticoagulants, and steroids to avoid reduced platelet function and ensure the treatment’s effectiveness [7]. Furthermore, the variety in platelet/leukocyte composition, PRP forms, and delivery methods in PRP research also determines its clinical applications [8].

Over the past years, several studies have compared the efficacy of IA-PRP to HA injections in patients with knee OA. A 1-year randomized clinical trial conducted by Raeissadat et al. reported that better results were determined in the PRP group compared with the HA group at the 12-month follow-up evaluated by WOMAC pain scores [9]. However, the presence of leukocytes in PRP remains controversial since it could affect the efficacy of knee OA treatment. Dragoo et al. found that leukocyte-rich platelet-rich plasma (LR-PRP) causes a significantly greater acute inflammatory response 5 days after injection compared with leukocyte-poor platelet-rich plasma (LP-PRP) in animal models [10]. However, some in vitro studies have reported that LR-PRP shows a higher level of growth factors and cytokines than LP-PRP [11]. Regarding the physiological effects of leukocytes in PRP preparations for knee OA treatments, further clinical studies still have to be conducted. Randomized controlled trials have been finished, reporting that LP-PRP treatment is better in terms of functional improvement and pain relief concerning HA treatment [12]; however, no meta-analysis has solely discussed the efficacy of knee IA LP-PRP injection as compared with HA. The purpose of our study is to investigate the efficacy and safety of intraarticular LP-PRP compared with HA injection for the treatment of knee OA. We hypothesize that intraarticular LP-PRP may offer superior clinical efficacy in improving pain relief and physical function compared with HA in patients with knee OA.

## Methods

This systematic review and meta-analysis was conducted on the basis of the recommendations of the Preferred Reporting Items for Systematic Reviews and Meta-Analyses Statement (PRISMA) [13] and the Cochrane Handbook for Systematic Reviews of Intervention [14]. No ethical approval and patient consent were required because this study is a systematic review of previously published RCTs.

### Search strategy

We systematically searched the included Web of Science, PubMed, Embase, and Cochrane Library trials. We used the keywords and MeSH terms “knee osteoarthritis,” “platelet-rich plasma,” “PRP,” “LP-PRP,” “leukocyte-poor,” “hyaluronic acid,” and “HA.” The included trials in our systemic review and meta-analysis were published between December 2012 and March 2021. Two investigators independently performed the initial searches, screened the titles and abstracts for selecting eligible RCTs, and examined the full articles. The reference lists of the studies were also scanned to search for additional studies. A third investigator reviewed all discrepancies, and the final decision on the included RCTs was determined by group consensus.

### Inclusion and exclusion criteria

Only RCTs were eligible for our meta-analysis, with an experimental group that received intraarticular LP-PRP injection and a control group that received intraarticular HA injection. RCTs were performed on adult humans (over 18 years of age) with osteoarthritis, and only studies published in English were included.

The exclusion criteria were as follows: (1) patients under 18 years of age; (2) studies that are non-RCT; (3) studies without a control group.

### Data extraction

Two authors independently extracted the following data from each trial: author, country of origin, publication year, study type, number of patients, age/gender, outcome measurements, and follow-up period. Injection doses, times, and intervals of LP-PRP and HA injections were also extracted. We extracted all data from tables or texts in original studies. A third investigator reviewed all discrepancies.

### Quality assessment

Two investigators independently used the method of the Cochrane risk of bias assessment scale [14] to evaluate each RCT. The method incorporates seven categories of bias: random selection, blinding of participants and outcome assessment, allocation concealment, reporting bias, outcome data, and other study biases. In each category, three levels (high risk, low risk, unclear risk) were summarized.

### Statistical analysis

The Review Manager 5.3 (Nordic Cochrane Center, Cochrane Collaboration, Copenhagen, Sweden) was used to conduct the systematic review and meta-analysis. Continuous variables were expressed as the mean and standard deviation (SD), and the treatment effects were expressed as mean difference (MD). The heterogeneity of individual studies was assessed by Higgins *I*^2^ statistic. A random-effects model was utilized if obvious heterogeneity existed (if *I*^2^ > 50% and *P* < 0.10); the fixed-effects model was used if no obvious heterogeneity existed (if *I*^2^ < 50%). All results were reported with 95% confidence intervals (CI), and a *P* value < 0.05 was considered to be of statistical significance. We also further performed subgroup analyses of the RCTs.

## Results

### Results of the search

**Fig. 1:**
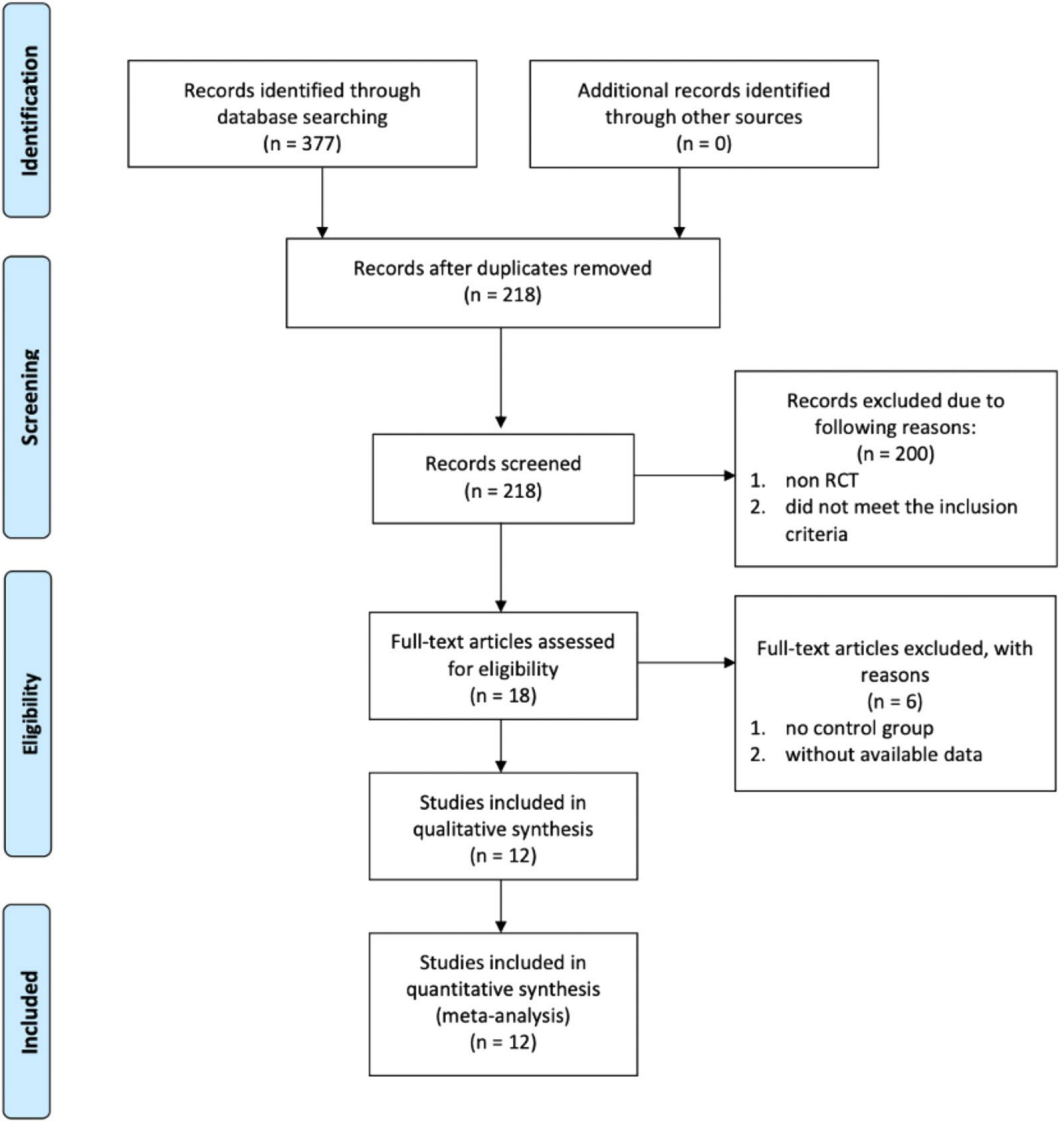
PRISMA flow chart of the study search and selection process

Figure 1 shows the literature selection progress. A total of 377 potentially relevant studies from PubMed, Web of Science, and Cochrane Library were yielded from the initial literature search. After 159 duplicated studies were removed, two authors independently screened the remaining 218 studies by scanning titles and reading abstracts. Subsequently, 200 studies were removed because these studies did not meet our inclusion criteria. We reviewed the full texts of the remaining 18 studies that had the potential for inclusion, and 6 of the studies were subsequently removed because no control groups were included or because data were not available. Ultimately, 12 RCTs were included in our systematic review and meta-analysis.

### Study characteristics

The study characteristics of the RCTs are presented in Table 1. These studies were published from 2012 to 2021. A total of 983 patients were included in our meta-analysis, with 12 RCTs included. Among these, 502 patients underwent LP-PRP injection and 481 patients underwent HA injection. Table 2 presents the timing and dosage of LP-PRP and HA injections. Among all of the included trials, four RCTs were conducted in Spain, three RCTs were conducted in China, and one RCT was conducted in Turkey, Iran, France, South USA, and Italy, respectively. The preinjection WOMAC scores and VAS scores are presented in Table 3. Three studies [15–17] did not report the preinjection WOMAC scores, and six studies [15, 17–20] did not report the preinjection VAS scores. Most studies revealed no statistical difference in preinjection WOMAC scores. The statistical results of seven studies [18, 20–25] revealed no significant differences (*P* > 0.05) in preinjection WOMAC scores between the LP-PRP and HA groups; and six studies [16, 22–26] revealed no significant differences (*P* > 0.05) in preinjection VAS scores between the LP-PRP and HA groups. The statistical results of the two studies [19, 26] revealed significant differences (*P* < 0.05) in preinjection WOMAC scores between the LP-PRP and HA groups. Most studies reported preinjection total WOMAC scores; however, one study [26] used the WOMAC pain score and one study [18] used the normalized total WOMAC score (scale from 1 to 100) as the measurements.

**Table 1:** The main characteristic of the included RCTs

Included trials	Study type	Group	Patients	Follow-up period (months)	Age (years, LP-PRP/HA)	Gender (male/female, *N*)	OA grades	Outcome measurements	Funding	Trial registration
Cerza et al., 2012 (Italy) [19]	RCT	LP-PRP HA	60 60	1, 3, 6	66.5 ± 11.3 66.2 ± 10.6	25/35 28/32	Kellgren–Lawrence classification 1–3	WOMAC	Not mentioned	Not mentioned
Sánchez et al., 2012 (Spain) [18]	RCT	PRGF HA	79 74	6	60.5 ± 7.9 58.9 ± 8.2	NA	Ahlbäck Grades 1–3	WOMAC, adverse effects	Not mentioned	Not mentioned
Say et al., 2012 (Turkey) [16]	RCT	LP-PRP HA	45 45	3, 6	55.2 ± 7.8 56.2 ± 5.1	5/40 6/39	Kellgren–Lawrence classification 2–3	VAS, adverse effects	Not mentioned	Not mentioned
Vaquerizo et al., 2013 (Spain) [21]	RCT	PRGF HA	48 48	6, 12	62.4 ± 6.6 64.8 ± 7.7	16/32 22/26	Kellgren–Lawrence classification 2–4	WOMAC, adverse effects	From the Departments of Orthopaedic Surgery and Clinical, Príncipe de Asturias University Hospital, Alcalá de Henares; Orthopaedic Surgery Department, Fundación García Cugat, Hospital Quirón Barcelona (R.S.), Barcelona; and BTI Biotechnology Institute ImasD, Vitoria, Spain	EudraCT number 2015-004738-90
Montañez-Heredia et al., 2016 (Spain) [17]	RCT	LP-PRP HA	27 26	3, 6	66.3 ± 8.3 61.5 ± 8.6	12/15 9/17	Kellgren–Lawrence classification 1–3	Adverse effects	Not mentioned	Not mentioned
Cole et al., 2017 (USA) [26]	RCT	LP-PRP HA	49 50	6, 12	55.9 ± 10.4 56.8 ± 10.5	28/21 20/30	Kellgren–Lawrence classification 2–4	WOMAC, VAS, IKDC	Aesculap/B. Braun, Arthrex, Athletico, Cytori, Medipost, National Institutes of Health, Ossur, Smith & Nephew, Tornier, and Arthrex and Kensey Nash	Not mentioned
Raeissadat et al., 2017 (Iran) [22]	RCT	PRGF HA	36 33	2, 3, 6	57.0 ± 7.18 59.5 ± 7.54	7/29 6/27	Kellgren–Lawrence classification 1–4	WOMAC, VAS	No funding	Iranian Registry of Clinical Trials (IRCT): IRCT2013121815860N1
Louis et al., 2018 (France) [23]	RCT	LP-PRP HA	24 24	1, 3, 6	53.2 ± 11.7 48.5 ± 11.5	14/10 11/13	Kellgren–Lawrence classification 1–4	WOMAC, VAS, adverse effects	No funding	ClinicalTrials.gov: NCT02211521
Buendía‐López et al., 2019 (Spain) [25]	RCT	LP-PRP HA	33 32	6, 12	56.15 ± 3.0 56.63 ± 2.9	16/17 15/17	Kellgren–Lawrence classification 1–2	WOMAC, VAS, adverse effects	No funding	NCT02990745
Huang et al., 2019 (China) [24]	RCT	LP-PRP HA	40 40	3, 6, 9, 12	54.5 ± 1.2 54.8 ± 1.1	25/15 19/21	Kellgren–Lawrence classification 1–2	WOMAC, VAS, adverse effects	Not mentioned	BTI-01-EC/07/ART
Lin et al., 2019 (Taiwan) [20]	RCT	LP-PRP HA	31 29	1, 2, 6, 12	61.17 ± 13.08 62.53 ± 9.9	9/22 10/19	Not mentioned	WOMAC, IKDC, adverse effects	Kaohsiung Veterans General Hospital Research Grant (VGHKS 103-075), and was registered with the Government Research Bulletin in Taiwan	PG10301-0457
Xu et al., 2021 (China) [15]	RCT	LP-PRP HA	30 20	1, 6, 12, 24	56.9 ± 4.2 57.1 ± 3.4	10/20 5/15	Kellgren–Lawrence classification 2–3	WOMAC, VAS	National Natural Science Foundation of China ( 51,773,098, 81,670,817, 81,970,772, 21,908,019 and 21,776,044), Natural Science Foundation of Tianjin City of China ( 18JCYBJC28300) and the Fundamental Research Funds for Central Universities (China)	ChiCTR-ONC-17013097

**Table 2:** The treatment protocols of LP-PRP and HA injections

Included trials	LP-PRP				HA			
	Injection dose (mL)	Intervals (weeks)	Times	Preparation methods and injection techniques	Injection dose	Intervals (weeks)	Times	Preparation methods and injection techniques
Cerza et al., 2012 (Italy) [19]	5.5	1	4	Autologous blood was drawn from patients, processed using a specific protocol to concentrate platelets, and then administered as 5.5 mL of autologous conditioned plasma	20 mg/2 mL	1	4	Commercially available product. Performed by an unblinded physician
Sánchez et al., 2012 (Spain) [18]	8	1	3	Autologous blood was drawn from patients, processed to concentrate platelets, and then injected in a series of 3 weekly sessions	NA	1	3	Commercially available product. Performed by an unblinded physician
Say et al., 2012 (Turkey) [16]	2.5	NA	1	Prepared from the patient’s autologous blood but not mentioned the process	25 mg/2.5 mL	1	3	Commercially available product, and injected under sterile conditions
Vaquerizo et al., 2013 (Spain) [21]	8	2	3	36 mL of blood was drawn from each patient, centrifuged at 580*g* for 8 min, and the PRGF-Endoret was separated from the red blood cells and leukocytes. The 2 mL PRGF-Endoret fractions were combined into 8 mL, activated with 400 µL calcium chloride, and injected as 8 mL into the joint	NA	NA	1	A single injection of Durolane (hyaluronic acid) was administered, which is a high-molecular-weight molecule synthesized via biofermentation using nonpathogenic *Streptococcus* bacteria and purified
Montañez-Heredia et al., 2016 (Spain) [17]	NS	2	3	18 mL of blood was collected and processed with double-spin centrifugation. The first spin separated plasma and red blood cells, and the second concentrated the platelets. The PRP was activated with calcium chloride or thrombin, and 3 mL was injected into the knee under sterile conditions	NA	2	3	Hylan G-F 20 (Synvisc), a commercially available hyaluronic acid was used, and injected under sterile conditions
Cole et al., 2017 (USA) [26]	4	1	3	Around 10 mL of blood was collected, centrifuged at 1500 rpm for 5 min to produce 4 mL of PRP. The PRP was processed and injected into the knee within 30 min, eliminating the need for anticoagulants	16 mg/ 2 ml	1	3	Commercially available product (Sanofi-Aventis) was administered
Raeissadat et al., 2017 (Iran) [22]	5	3	2	PRP was processed with the Rooyagen Kit. After drawing 35–40 mL of blood and adding 5 mL of anticoagulant, the blood was centrifuged at 1600 rpm for 15 min to separate the layers. The plasma and buffy coat were then centrifuged at 2800 rpm for 7 min, yielding 4–6 mL of PRP with leukocytes	20 mg	1	3	Commercially available product (Hyalgan, Fidia Farmaceutici S.p.A., Abano Terme, Italy) was administered. Performed by an unblinded physician
Louis et al., 2018 (France) [23]	3	NA	1	30 mL of blood was drawn and mixed with 3 mL of acid-citrate-dextrose. After centrifugation at 1500 rpm for 10 min, the plasma layer containing concentrated platelets was collected for injection	60 mg/ 3 ml	NA	1	Hylan G-F 20 (Synvisc), a commercially available hyaluronic acid was used
Buendía‐López et al., 2019 (Spain) [25]	5	NA	NA	18 mL of blood was collected, centrifuged twice at 1800 rpm and 3500 rpm to concentrate platelets. The PRP was activated with calcium chloride or thrombin, then 3 mL was injected into the knee under sterile conditions	60 mg/ 2 ml	NA	NA	Hylan G-F 20 (Synvisc), a commercially available hyaluronic acid was used
Huang et al., 2019 (China) [24]	2	1	3	8 mL of blood was collected from the cubital vein and centrifuged for 5 min at either 1500 g or 3500 rpm, based on manufacturer recommendations. This process utilized a single centrifugation step, which separated blood components into layers. Erythrocytes settled at the bottom, followed by a buffy coat of white blood cells, and platelets concentrated just above the buffy coat within the plasma	4 ml (500–730 kDa)	1	3	A commercially available hyaluronic acid was used (SK chemical research Co., Ltd., Tokyo, Japan). Performed by an unblinded physician
Lin et al., 2019 (Taiwan) [20]	5	1	3	PRP was prepared using RegenKit-THT, where 10 mL of blood was drawn and centrifuged at 1500 rpm for 8 min. This yielded about 5.0 ± 0.5 mL of PRP, with a platelet concentration of 1.81 ± 0.34 times the baseline. The product was leukocyte poor, as nearly 70% of white blood cells were removed during centrifugation	20 mg/ 2 ml	1	3	A commercially available hyaluronic acid was used. Performed by an unblinded physician
Xu et al., 2021 (China) [15]	4	2	3	A 36-mL blood sample was collected and mixed with 4 mL of acid citrate dextrose, then centrifuged at 160*g* for 10 min to separate components. Platelet-containing plasma was transferred, centrifuged again at 250*g* for 15 min, and the resulting leukocyte-poor PRP was collected using a 5-mL syringe	20 mg/ 2 ml	2	3	A commercially available hyaluronic acid was used. Performed by an unblinded physician

NA: not applicable

**Table 3:** Preinjection WOMAC scores and VAS scores

Study	Preinjection WOMAC total scores			Preinjection VAS scores		
	LP-PRP	HA	*P* value	LP-PRP	HA	*P* value
Cerza, 2012 (Italy) [19]	79.6 ± 9.5	75.4 ± 10.7	0.025	N/A	N/A	N/A
Sánchez, 2012 (Spain) [18]	121.8 ± 44.4 (†Normalized WOMAC scale)	115.6 ± 45.1 (†Normalized WOMAC scale)	0.378	N/A	N/A	N/A
Say, 2012 (Turkey) [16]	N/A	N/A	N/A	7.3 ± 1.6	7 ± 1.3	0.234
Vaquerizo, 2013 (Spain) [21]	45.9 ± 12.7	50.8 ± 18.4	0.137	N/A	N/A	N/A
Montañez-Heredia, 2016 (Spain) [17]	N/A	N/A	N/A	N/A	N/A	N/A
Cole, 2017 (USA) [26]	7 ± 0.53 (WOMAC pain score)	7.52 ± 0.58 (WOMAC pain score)	0.0001	5.72 ± 1.43	6.29 ± 1.57	0.0619
Raeissadat, 2017 (Iran) [22]	42.9 ± 13.5	38.8 ± 12.6	0.197	7.8 ± 1.78	7.4 ± 1.48	0.316
Louis, 2018 (France) [23]	35.5 ± 15.5	32.5 ± 23.1	0.599	4.8 ± 2.3	5.0 ± 2.4	0.712
Buendía‐López, 2019 (Spain) [25]	42.57 ± 7.3	42.62 ± 7.3	0.978	6.15 ± 1.1	6.06 ± 0.9	0.72
Huang, 2019 (China) [24]	48.19 ± 4.96	47.23 ± 5.37	> 0.05	4.57 ± 0.61	4.54 ± 0.6	0.825
Lin, 2019 (Taiwan) [20]	52.8 ± 18.1	52.7 ± 18.1	0.601	N/A	N/A	N/A
Xu, 2021 (China) [15]	N/A	N/A	N/A	N/A	N/A	N/A

*N/A* not applicable, due to no data provided from the original research

†Normalized scores for the WOMAC can range from 0 to 100 for all subscales

### Risk of bias

**Fig. 2:**
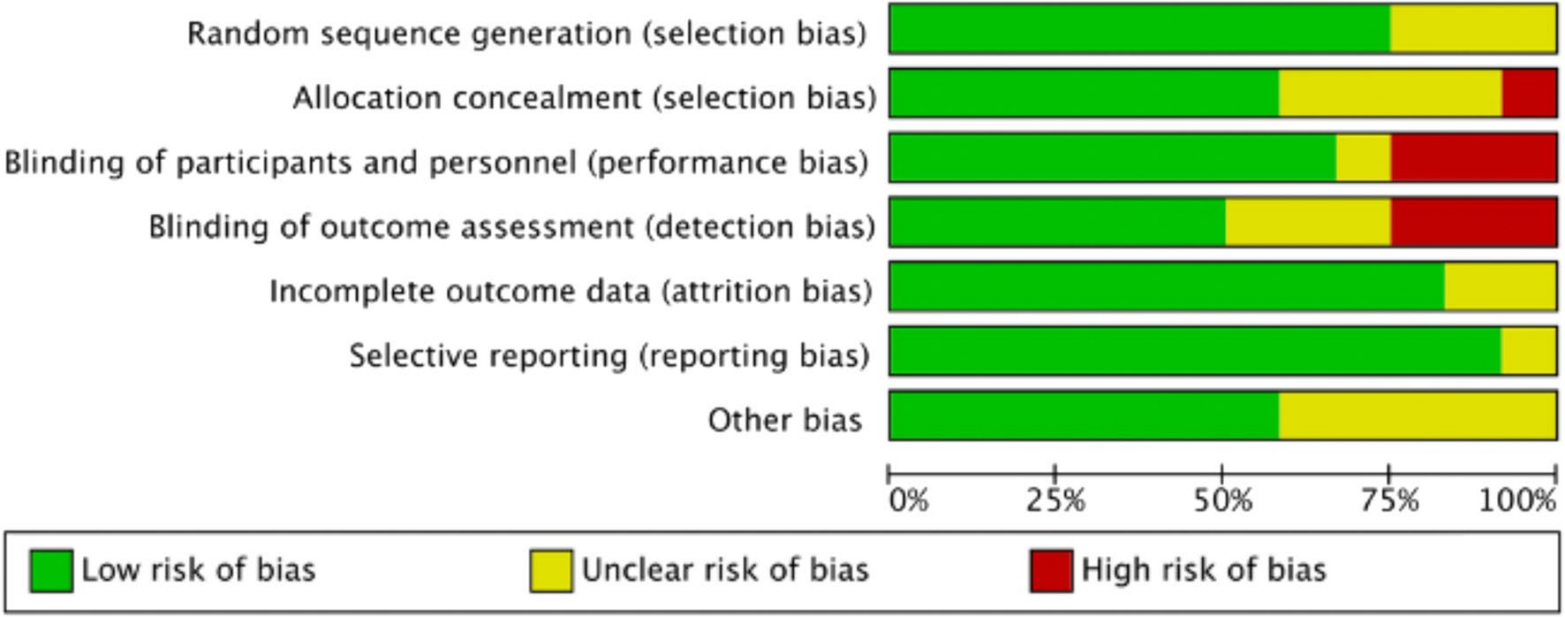
Risk-of-bias graph

**Fig. 3:**
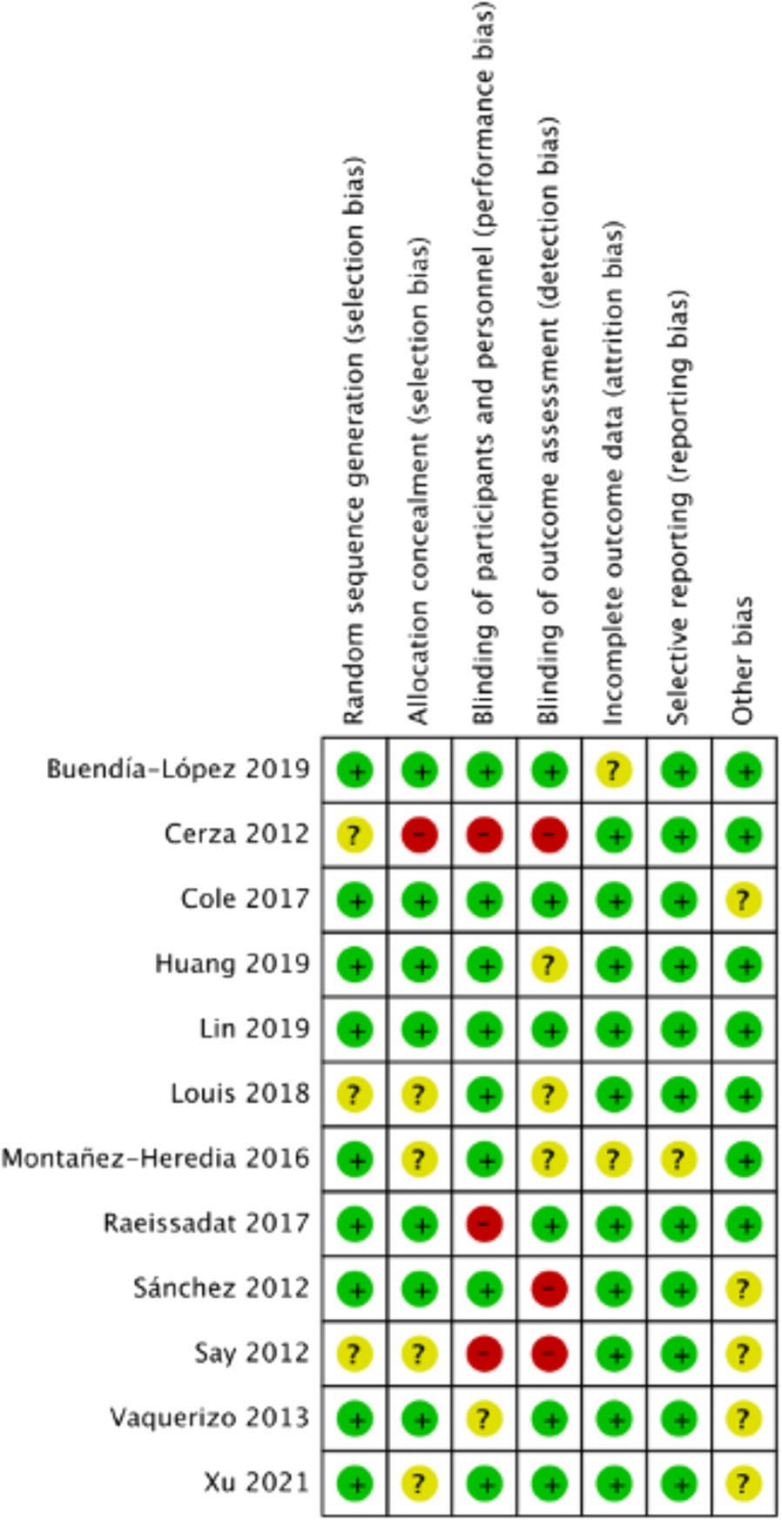
Risk-of-bias summary

Figures 2 and 3 reveal the risk bias summary and graph of the included trials. Among all RCTs, the methods of random sequence generation were not reported in three studies [16, 19, 23]. Allocation concealment was recorded in seven studies [18, 20–22, 24–26]. Four studies [15, 20, 25, 26] were double-blinded. Eight studies [15, 17, 18, 20, 23–26] reported blinding of participants and personnel, and six studies [15, 20–22, 25, 26] reported blinding of outcome assessors.

### WOMAC total scores

**Fig. 4:**
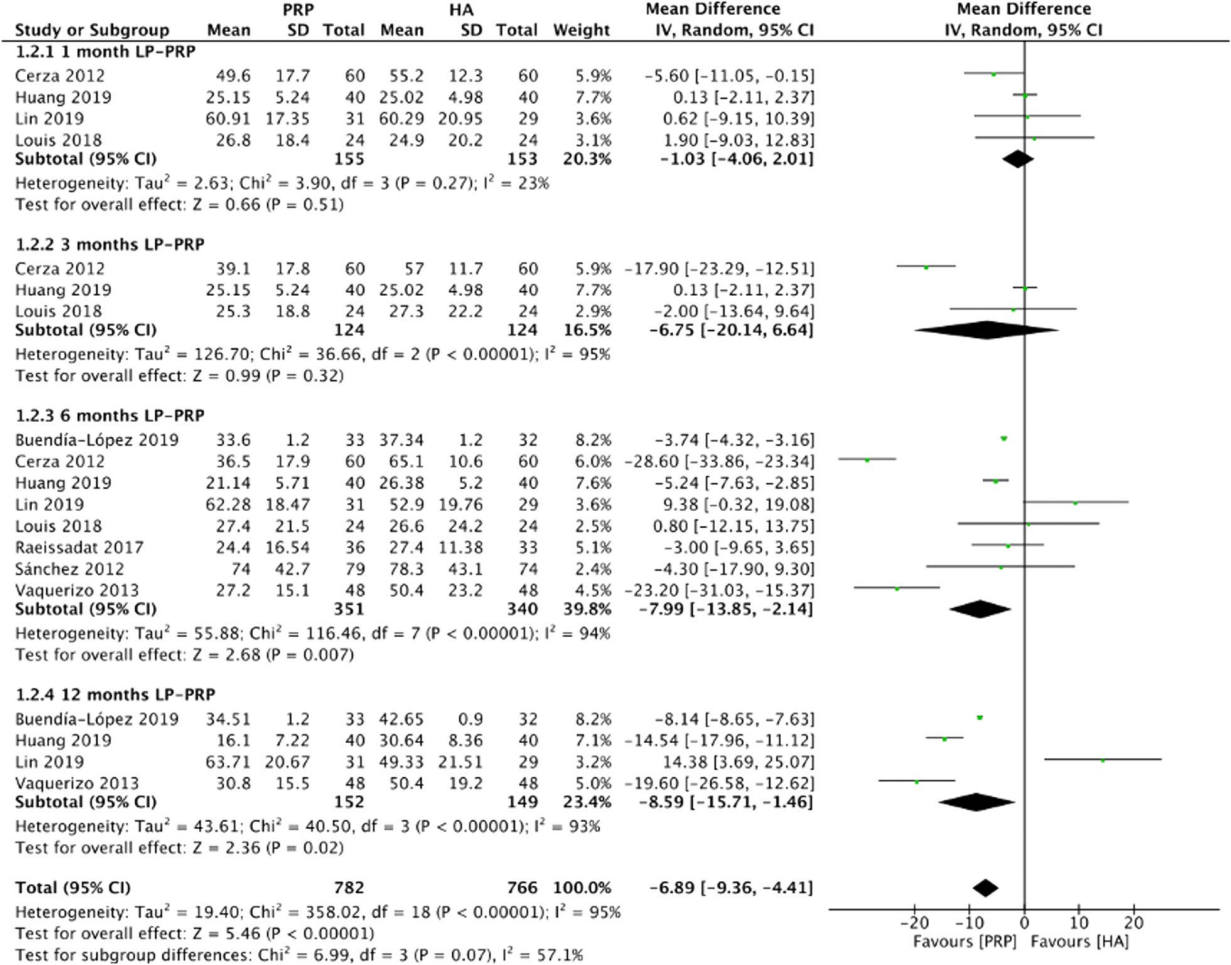
Forest plot for WOMAC total scores between LP-PRP and HA groups. *IV* inverse variance, *CI* confidence interval, *SD* standard deviation

Figure 4 summarizes the WOMAC total scores comparing intraarticular LP-PRP and HA injection. Due to the heterogeneity between included trials being significant (*I*^2^ = 95%, *P* < 0.00001), a random-effect model was used. The pooled results showed that the intraarticular LP-PRP injection was associated with a lower WOMAC total score compared with HA injection (MD −6.89, 95% CI −9.36 to −4.41, *P* < 0.00001). Four studies [19, 20, 23, 24] reported WOMAC total scores at 1 month post-treatment (*I*^2^ = 23%, MD −1.03, 95% CI −4.06 to 2.01, *P* = 0.32); three studies [19, 23, 24] reported WOMAC total scores at 3 months post-treatment (*I*^2^ = 95%, MD −6.75, 95% CI −20.14 to 6.64, *P* = 0.32); eight studies [18–25] reported WOMAC total scores at 6 months post-treatment (*I*^2^ = 79%, MD −7.99, 94% CI −13.85 to −2.14, *P* = 0.007); and four studies [20, 21, 24, 25] reported WOMAC total scores at 12 months post-treatment (*I*^2^ = 93%, MD −8.59, 95% CI −15.71 to −1.46, *P* = 0.02). The subgroup analysis showed that the WOMAC total scores of the LP-PRP group were statistically significantly lower at 6 and 12 months after treatment, compared with the HA group.

### WOMAC pain scores

**Fig. 5:**
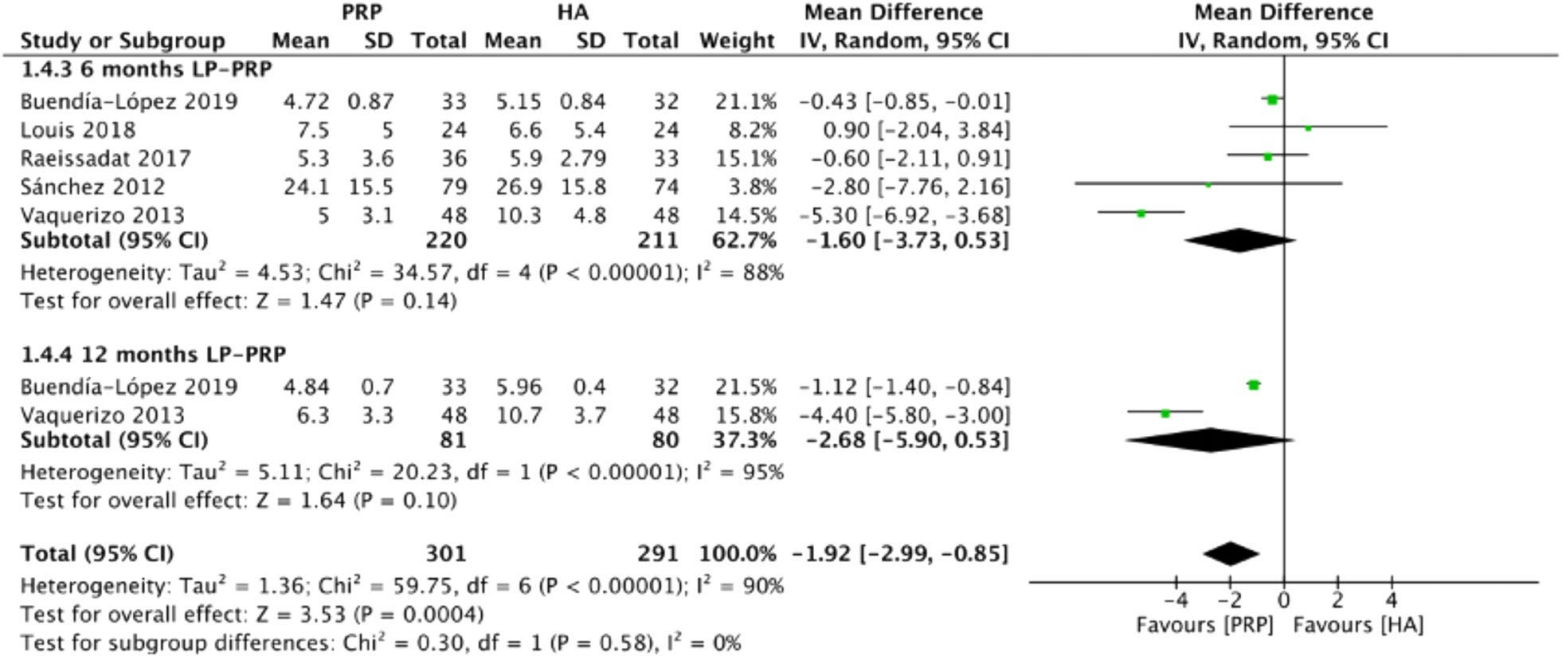
Forest plot for WOMAC pain scores between LP-PRP and HA groups. *IV* inverse variance, *CI* confidence interval, *SD* standard deviation

Figure 5 summarizes the WOMAC pain scores comparing intraarticular LP-PRP and HA injection. Due to the heterogeneity between included trials being significant (*I*^2^ = 90%, *P* < 0.00001), a random-effect model was used. The pooled results showed that the intraarticular LP-PRP injection was associated with a lower WOMAC pain score compared with HA injection (MD −1.92, 95% CI −2.99 to −0.85, *P* = 0.0004). Five studies [18, 21–23, 25] reported WOMAC pain scores at 6 months post-treatment (*I*^2^ = 88%, MD −1.6, 95% CI −3.73 to 0.53, *P* = 0.14); two studies [21, 25] reported WOMAC pain scores at 12 months post-treatment (*I*^2^ = 95%, MD −2.68, 95% CI −5.9 to 0.53, *P* = 0.1). The subgroup analysis results demonstrated that the WOMAC pain scores of the LP-PRP group showed no significance at 6 and 12 months after treatment, compared with the HA group.

### WOMAC stiffness scores

**Fig. 6:**
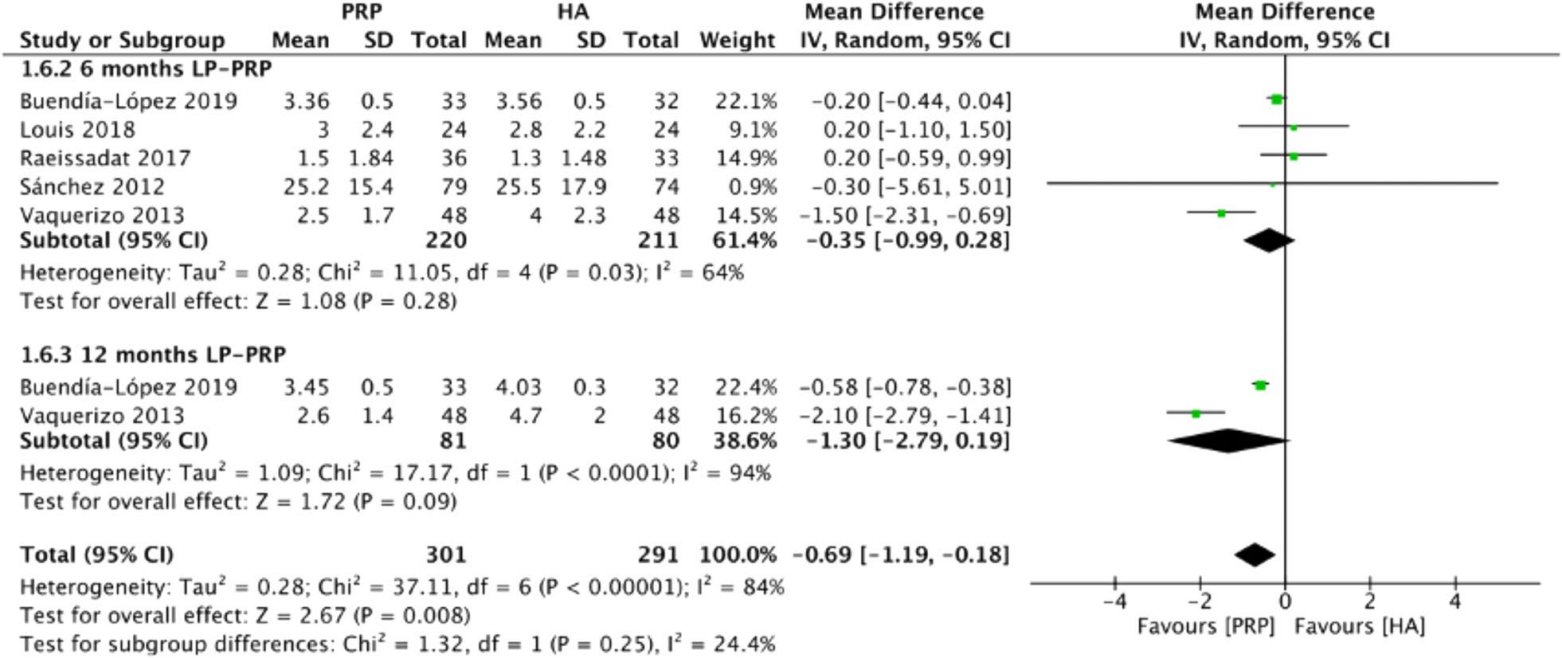
Forest plot for WOMAC stiffness scores between LP-PRP and HA groups. *IV* inverse variance, *CI* confidence interval, *SD* standard deviation

Figure 6 summarizes the WOMAC stiffness scores comparing intraarticular LP-PRP and HA injection. Due to the heterogeneity between included trials being significant (*I*^2^ = 84%, *P* < 0.00001), the random-effect model was used. The pooled results showed that the LP-PRP injection was associated with a lower WOMAC stiffness scores compared with the HA injection (MD −0.69, 95% CI −1.19 to −0.18, *P* = 0.008). Five studies [18, 21–23, 25] reported WOMAC stiffness scores at 6 months post-treatment (*I*^2^ = 64%, MD −0.35, 95% CI −0.99 to 0.28, *P* = 0.28); two studies [21, 25] reported WOMAC stiffness scores at 12 months post-treatment (*I*^2^ = 94%, MD −1.3, 95% CI −2.79 to 0.19, *P* = 0.09). The subgroup analysis demonstrated that the WOMAC stiffness scores of the LP-PRP group showed no significance at 6 and 12 months after treatment, compared with the HA group.

### WOMAC physical function scores

**Fig. 7:**
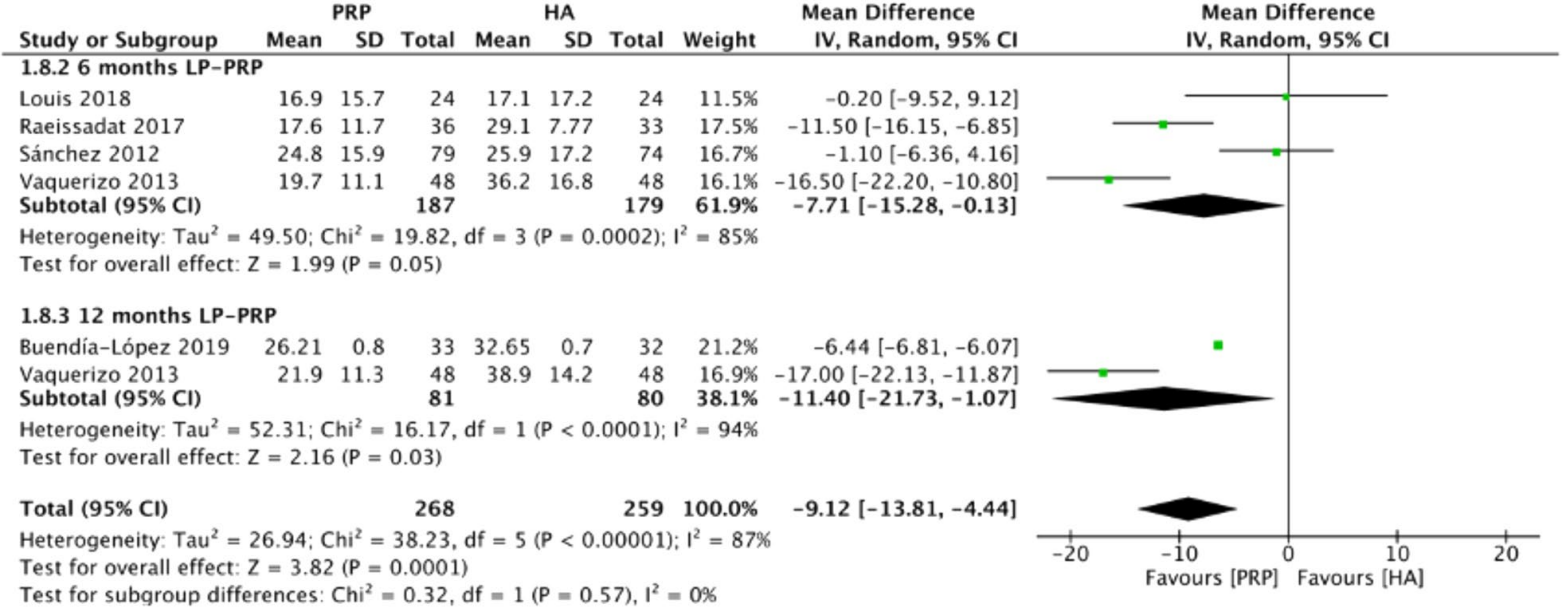
Forest plot for WOMAC physical function scores between LP-PRP and HA groups. *IV* inverse variance, *CI* confidence interval, *SD* standard deviation

Figure 7 summarizes the WOMAC physical function scores comparing intraarticular LP-PRP and HA injection. Due to the heterogeneity between included trials being significant (*I*^2^ = 87%, *P* < 0.00001), the random-effect model was used. The pooled results showed that the LP-PRP injection was associated with lower WOMAC physical function scores than HA injection (MD −9.12, 95% CI −13.81 to −4.44, *P* = 0.0001). Four studies [18, 21–23] reported WOMAC physical function scores at 6 months post-treatment (*I*^2^ = 85%, MD −7.71, 95% CI −15.28 to −0.13, *P* = 0.05); two studies [21, 25] reported WOMAC physical function scores at 12 months post-treatment (*I*^2^ = 94%, MD −11.4, 95% CI −21.73 to −1.07, *P* = 0.03). The subgroup analysis demonstrated that the WOMAC physical function scores of the LP-PRP group were statistically significantly lower at 12 months after treatment, compared with the HA group.

### VAS score

**Fig. 8:**
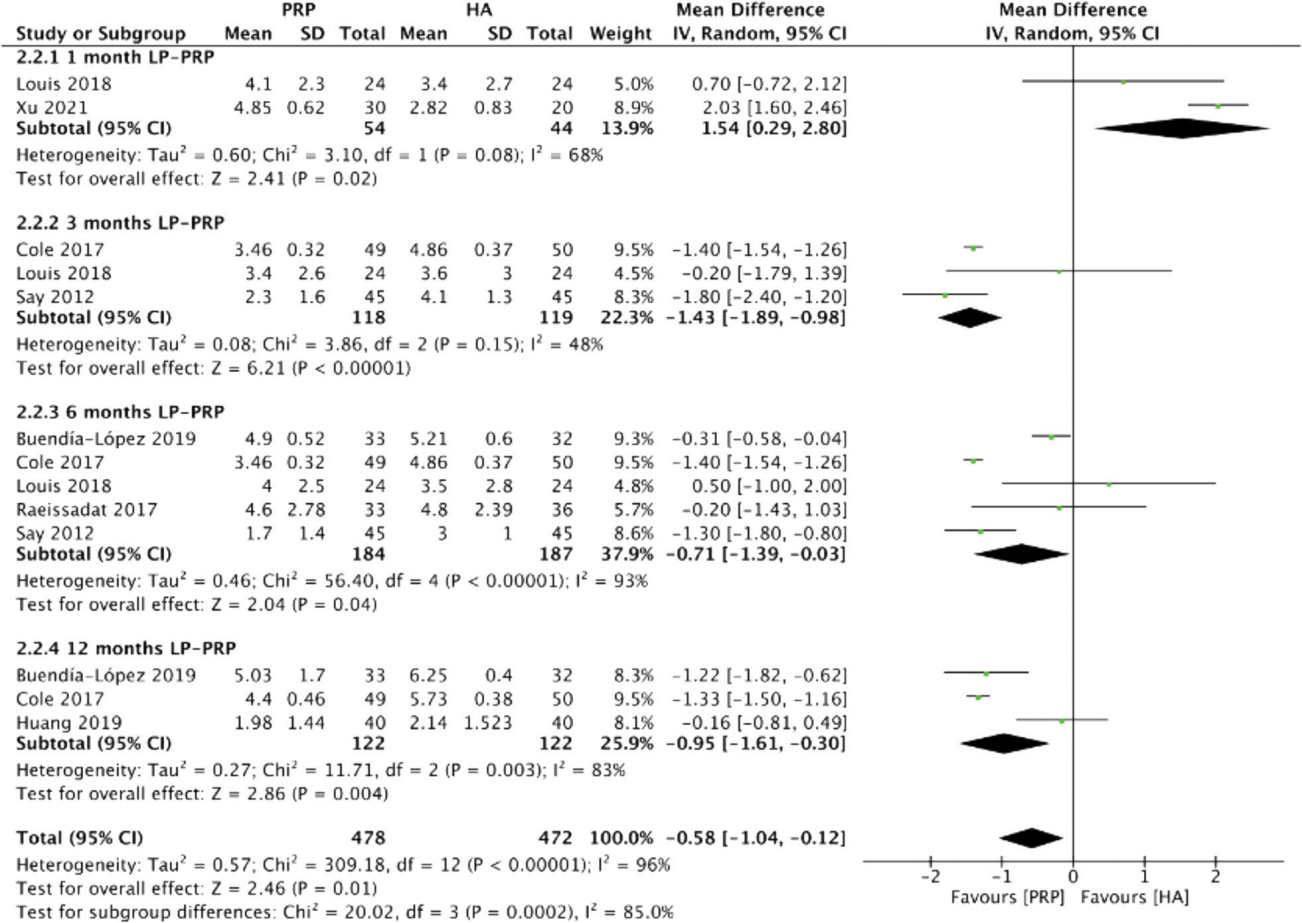
Forest plot for VAS scores between LP-PRP and HA groups. *IV* inverse variance, *CI* confidence interval, *SD* standard deviation

Figure 8 summarizes the VAS scores comparing intraarticular LP-PRP and HA injection. Due to the heterogeneity between included trials being significant (*I*^2^ = 96%, *P* < 0.00001), the random-effect model was used. The pooled results showed that the LP-PRP injection was associated with a lower VAS score compared with HA injection (MD −0.58, 95% CI −1.04 to −0.12, *P* = 0.01). Two studies [15, 23] reported VAS scores at 1 month post-treatment (*I*^2^ = 68%, MD 1.54, 95% CI 0.29 to 2.8, *P* = 0.02); three studies [16, 23, 26] reported VAS scores at 3 months post-treatment (*I*^2^ = 48%, MD −1.43, 95% CI −1.89 to −0.98, *P* < 0.00001); five studies [16, 22, 23, 25, 26] reported VAS scores at 6 months post-treatment (*I*^2^ = 93%, MD −0.71, 95% CI −1.39 to −0.03, *P* = 0.04); and three studies [24–26] reported VAS scores at 12 months post-treatment (*I*^2^ = 83%, MD −0.95, 95% CI −1.61 to −0.3, *P* = 0.004). The subgroup analysis demonstrated that LP-PRP injection had a better effect on pain relief than those with HA injection at 3, 6, and 12 months post-treatment, and HA injection had better pain relief than those with LP-PRP injection at 1 month post-treatment.

### IKDC score

**Fig. 9:**
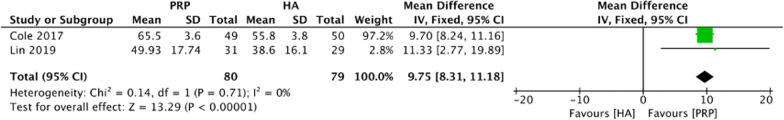
Forest plot for IKDC score between LP-PRP and HA groups. *IV* inverse variance, *CI* confidence interval, *SD* standard deviation

Figure 9 summarizes the IKDC score comparing intraarticular LP-PRP and HA injection at 6 months after treatment. Because heterogeneity between included trials was low (*I*^2^ = 0%, *P* = 0.71), the fixed-effect model was used. Two studies [20, 26] reported IKDC scores at 6 months post-treatment (*I*^2^ = 0%, MD 9.75, 95% CI 8.31 to 11.18, *P* < 0.00001). The IKDC score of the LP-PRP group compared with the HA group was significantly higher at 6 months after treatment.

### Adverse events

**Fig. 10:**
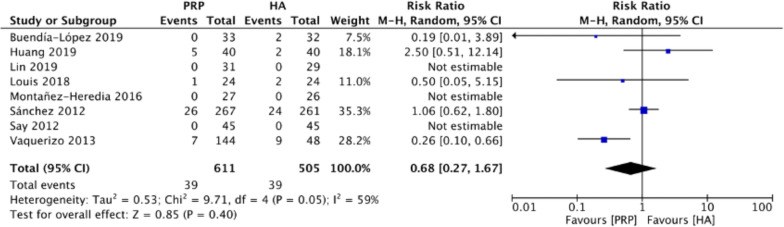
Forest plot for adverse effects between LP-PRP and HA groups. *M-H* Mantel–Haenszel, *CI* confidence interval

Figure 10 summarizes the adverse effects of the LP-PRP and HA groups on knee osteoarthritis. Eight RCTs [16–18, 20, 21, 23–25] were included. The random-effect model was used because the heterogeneity test showed moderate heterogeneity (*I*^2^ = 59%). No significant complications were reported. The results demonstrated no significant difference between the LP-PRP and HA groups (relative risk (RR) 0.68, 95% CI 0.27 to 1.67, *P* = 0.4). The result indicated that LP-PRP and HA had similar safety profiles.

## Discussion

The incidence of knee osteoarthritis has notably escalated owing to the upward trend in life expectancy [27]. Intraarticular injections of LP-PRP and HA have garnered substantial attention as nonoperative modalities for managing knee osteoarthritis. This meta-analysis involved a systematic review encompassing 12 randomized RCTs to assess the effectiveness of intraarticular LP-PRP and HA in the treatment of knee osteoarthritis. The findings demonstrated a significantly better improvement in both WOMAC total scores and WOMAC physical function scores at the 6- and 12-month intervals following treatment with LP-PRP, in contrast to the HA group. At 6 months post-injection, the LP-PRP group exhibited significantly superior IKDC scores compared with the HA group. Moreover, VAS scores were consistently superior in the LP-PRP group at 3, 6, and 12 months. Most importantly, there was no significant variance in adverse events between the two groups. However, we observed a discrepancy in subgroup analysis, where VAS pain scores showed no significant difference between the LP-PRP and HA groups, while WOMAC pain scores indicated a significant difference. This may stem from differences in methodology: the WOMAC pain score assesses pain across multidimensional daily activities, whereas the VAS pain score captures overall pain intensity at a single moment, leading to variability in pain assessment.

Previous systematic reviews and meta-analyses have extensively examined the therapeutic effects of PRP and HA in the management of knee OA. Dong et al. [28] compared the efficacy of intraarticular PRP with other injection modalities, including HA, saline, and prolotherapy. Their findings indicated superior outcomes with intraarticular PRP administration. Similarly, Duymus et al. [29] investigated the efficacy of PRP injections versus HA in patients with knee OA, demonstrating that PRP yielded superior therapeutic benefits, particularly in cases of mild-to-moderate knee OA. In addition, Lin et al. [20] conducted a comparative analysis of PRP and HA treatments for knee OA, highlighting the efficacy of LP-PRP in enhancing functional recovery for at least 1 year post-treatment. Our meta-analysis showed that WOMAC total scores and WOMAC physical function scores of the LP-PRP group were better than the HA group at 6 and 12 months. The strength of this study lies in being the first meta-analysis that specifically addresses the efficacy of knee intraarticular LP-PRP injections in comparison with HA. Furthermore, this paper includes the most RCTs on this topic, utilizing high-quality RCTs for the meta-analysis to substantiate the clinical benefits of LP-PRP.

Belk et al. [30] investigated 18 RCTs to examine the effectiveness of PRP injection in improving clinical outcomes compared with HA interventions. Their analysis revealed a significant improvement in clinical outcomes associated with PRP administration in contrast to HA treatments. Furthermore, through a pooled analysis of studies comparing LR-PRP and LP-PRP, no notable differences were observed in terms of WOMAC or VAS scores. However, the findings suggested a potential superiority of LP-PRP over LR-PRP concerning IKDC scores. Our study also demonstrated that the LP-PRP group exhibited superior outcomes in terms of IKDC scores compared with the HA group.

The optimal composition of LP-PRP for knee OA treatment remains contentious. Certain studies have indicated that LP-PRP outperforms LR-PRP in OA treatment [31]. This could be attributed to the enhanced anti-inflammatory properties of LP-PRP [31]. A meta-analysis [32] examined the impact of leukocyte concentration on the efficacy of PRP in the treatment of patients with knee OA. The study revealed that LP-PRP may yield superior functional outcome scores compared with LR-PRP. Notably, LP-PRP exhibited a significantly greater improvement in WOMAC scores compared with both placebo HA, whereas LR-PRP did not demonstrate such improvement. Furthermore, the leukocyte concentration of PRP was found to not affect the incidence of adverse reactions. Recent studies further examined the role of leukocytes in platelet-rich plasma treatments for knee osteoarthritis. A double-blind randomized controlled trial found that leukocyte presence in PRP did not affect treatment safety or efficacy [33]. Similarly, a network meta-analysis concluded that varying leukocyte concentrations in PRP injections did not significantly influence clinical outcomes for patients with knee OA [34]. Both studies suggest that leukocyte concentration in PRP may not be a critical factor in managing knee osteoarthritis.

There are some limitations to this study. Firstly, a notable proportion of our analyses displayed significant heterogeneity. Despite our efforts to address this through subgroup analyses, some results still exhibit substantial heterogeneity. This variability may be attributed to variations among patients, including discrepancies in age and gender, study design between studies, and the differences in LP-PRP injection techniques and PRP dosages across physicians and studies. We utilized subgroup analyses to further investigate the following categories: WOMAC total scores, WOMAC pain scores, WOMAC stiffness scores, WOMAC physical function scores, and VAS scores. The basis for subgroup classification was determined by the time of post-treatment assessment using the aforementioned scales. Secondly, due to the absence of data regarding prior treatments patients may have undergone before receiving LP-PRP or HA injections, we are unable to confirm whether all studies started with consistent baseline conditions across the samples. Thirdly, the relatively small sample sizes in some of the RCTs limited the statistical power of our study. Lastly, all the RCTs included in this meta-analysis were published in English, potentially introducing selection bias.

## Conclusion

Intraarticular LP-PRP injection demonstrated superior overall efficacy compared with HA injection among patients with knee OA, as indicated by significant improvements in WOMAC total scores, WOMAC physical function scores, VAS pain scores, and IKDC scores at 6- and 12-month follow-ups.

## Data Availability

All data generated or analyzed during this study are included in this published article.

## Abbreviations

CI: Confidence intervals
IA: Intraarticular
IKDC: International Knee Documentation Committee
IV: Inverse variance
HA: Hyaluronic acid
LP-PRP: Leukocyte-poor platelet-rich plasma
LR-PRP: Leukocyte-rich platelet-rich plasma
MD: Mean difference
NSAIDs: Nonsteroidal anti-inflammatory drugs
OA: Osteoarthritis
RCT: Randomized controlled trials
SD: Standard deviation
WOMAC: Western Ontario and McMaster Universities Arthritis Index
VAS: Visual analog scale
